# Aripiprazole: A Case for Side Effect Awareness

**DOI:** 10.1192/bjo.2025.10744

**Published:** 2025-06-20

**Authors:** Mario Lepore

**Affiliations:** South London and Maudsley NHS Foundation Trust, London, United Kingdom

## Abstract

**Aims:** Aripiprazole, an atypical antipsychotic, is commonly prescribed for various psychiatric conditions including schizophrenia and bipolar disorder. It is generally considered to have a more favourable side effect profile compared with other antipsychotics, with a lower risk of metabolic side effects and hyperprolactinemia. We present the case of a 57-year-old male who developed marked hypersexuality and excessive spending following the initiation of oral aripiprazole.

**Methods:** Mr X was referred for psychiatric review within our drug and alcohol service due to concerns regarding worsening paranoid ideation. He had a history of alcohol dependency but had been abstinent for five months at the time of referral. He denied recreational drug use and was not on regular medication, aside from thiamine 100 mg three times daily. His past medical history was unremarkable, though he reported a family history of schizophrenia, with a brother diagnosed with the condition. Mental state examination revealed a complex paranoid delusional system, accompanied by auditory hallucinations, thought interference and somatic passivity. Blood tests were unremarkable, and a urine drug screen was negative. Aripiprazole was prescribed and titrated to 15 mg daily over a two-week period, with potential risks, including those of disinhibition, being discussed. At follow-up, Mr X recalled the discussion and reported a significant increase in his libido, spending over £1500 over the course of a week on online sexualised adult chat websites, a behaviour he had never previously engaged in. The aripiprazole was switched to olanzapine and the hypersexuality resolved over the following four weeks, with no further excessive spending.

**Results:** Whilst generally well-tolerated, aripiprazole is not without potential side effects, including issues with impulse control, such as hypersexuality and excessive spending. These behaviours are thought to arise from aripiprazole’s partial agonist activity at dopamine D2 receptors. Given the significant financial and social consequences these behaviours can have, it is essential for clinicians to proactively discuss the possibility of these side effects and ensure close monitoring, particularly during the early stages of treatment or following dose adjustments.

**Conclusion:** Whilst the appropriate prescribing of antipsychotics forms a key part of many treatment plans, this case report serves as an important reminder of the potential rare but significant side effects of aripiprazole. Clinicians must remain vigilant for these behaviours and proactively discuss them with patients, who otherwise may feel reluctant in doing so. It is essential to provide clear warnings and ensure appropriate follow-up to address any emerging side effects.

